# Surgical interventions for Bertolotti’s syndrome: case report and review of unsatisfactory cases in the literature

**DOI:** 10.1186/s12893-022-01498-y

**Published:** 2022-02-02

**Authors:** Chun-Jen Chang, You-Pen Chiu, Hui-Ru Ji, Chang-Hung Chu, Cheng-Di Chiu

**Affiliations:** 1 Department of Neurosurgery, China Medical University Hospital, No.2, Yu-Der Road, North Area, Taichung, 40447 Taiwan, ROC; 2 Spine Center, China Medical University Hospital, Taichung, Taiwan; 3 Graduate Institute of Biomedical Science, China Medical University, Taichung, Taiwan; 4 School of Medicine, China Medical University, Taichung, Taiwan; 5 Department of Emergency, China Medical University Hospital, Taichung, Taiwan

**Keywords:** Bertollotti’s syndrome, Lumbosacral transitional vertebrae, Low back pain, Minimally invasive surgery, Case report

## Abstract

**Background:**

Bertolotti’s syndrome (BS) is characterized by the enlargement of transverse processes in caudal lumbar segments, causing chronic and persistent low back pain or sciatica. The present study aimed to describe our surgical technique for BS treatment and to review existing literature describing unsatisfactory outcomes.

**Case presentation:**

We report a patient who complained of lower back pain and soreness that intermittently radiated to his left leg for longer than 10 years. Based on an examination of his symptoms and signs, including imaging and electrical physiology assessments, in addition to the report of temporary pain relief after a local steroid injection to a diagnostic trigger site (articulation between the transverse process and the sacral ala or iliac crest), the patient was diagnosed with BS. The applied surgical techniques included minimally invasive, three-dimensional C-arm guidance, a tubing approach, and microscopic and bone cavitron ultrasonic surgical aspirator–assisted operations. After surgery, improvements were reported on the visual analog scale (from 8 to 2 points on a 10-point scale) and the straight leg raising test for the left leg (20° to 90°) within 3 days. The patient gained an ordinary life and returned to work within one week of surgery, with no observed postoperative complications.

**Conclusions:**

Minimally invasive tubular microscopic decompression and disarticulation is an effective method for treating BS. However, to achieve a successful outcome, it is recommended to use physician-proficient techniques that are in line with the patient’s diagnosis.

## Background

Low back pain (LBP) is a common condition resulting from various etiologies. One unique cause of LBP is the presence of lumbosacral transitional vertebrae (LSTV), referred to as Bertolotti’s syndrome (BS), first described by Dr. Mario Bertolotti [1]. A typical anatomical variation in BS presentation includes the occurrence of a transverse mega-apophysis, which may articulate or fuse with the iliac crest or sacrum. The prevalence of such congenital abnormalities has been reported at 4–35% among the total population, with an average incidence of 12.3% [2]. Approximately 11.4% of BS patients are diagnosed prior to the age of 30 years [3].

LSTVs can be classified into Types I–IV. Type I is the most common type but is less likely to be a source of LBP compared with other types of LSTV [2, 4]. The underlying mechanisms through which LSTVs cause LBP remain controversial. Previous studies have demonstrated that symptomatic LSTVs may be associated with the pathology of the intervertebral disc, spinal canal stenosis, extraforaminal stenosis, or facet joint arthrosis [5–8]. Reported therapeutic approaches for BS vary, including local injections of anesthesia or steroids into the pseudoarticulation space; radiofrequency coagulation; and surgical interventions, such as the resection of the pseudoarticulation, decompression of the extraforaminal entrapped nerve root, and spinal fusion [9–14]. In the present study, we reported a case who suffered from persisted LBP and was diagnosed with BS. A microscopic LSTV resection was performed via a minimally invasive, tubular approach with the assistance of a bone cavitron ultrasonic surgical aspirator (CUSA). We report the technique and attempt to identify the underlying reasons for unsatisfactory therapeutic results reported in the existing literature.

## Case presentation

**Fig. 1:**
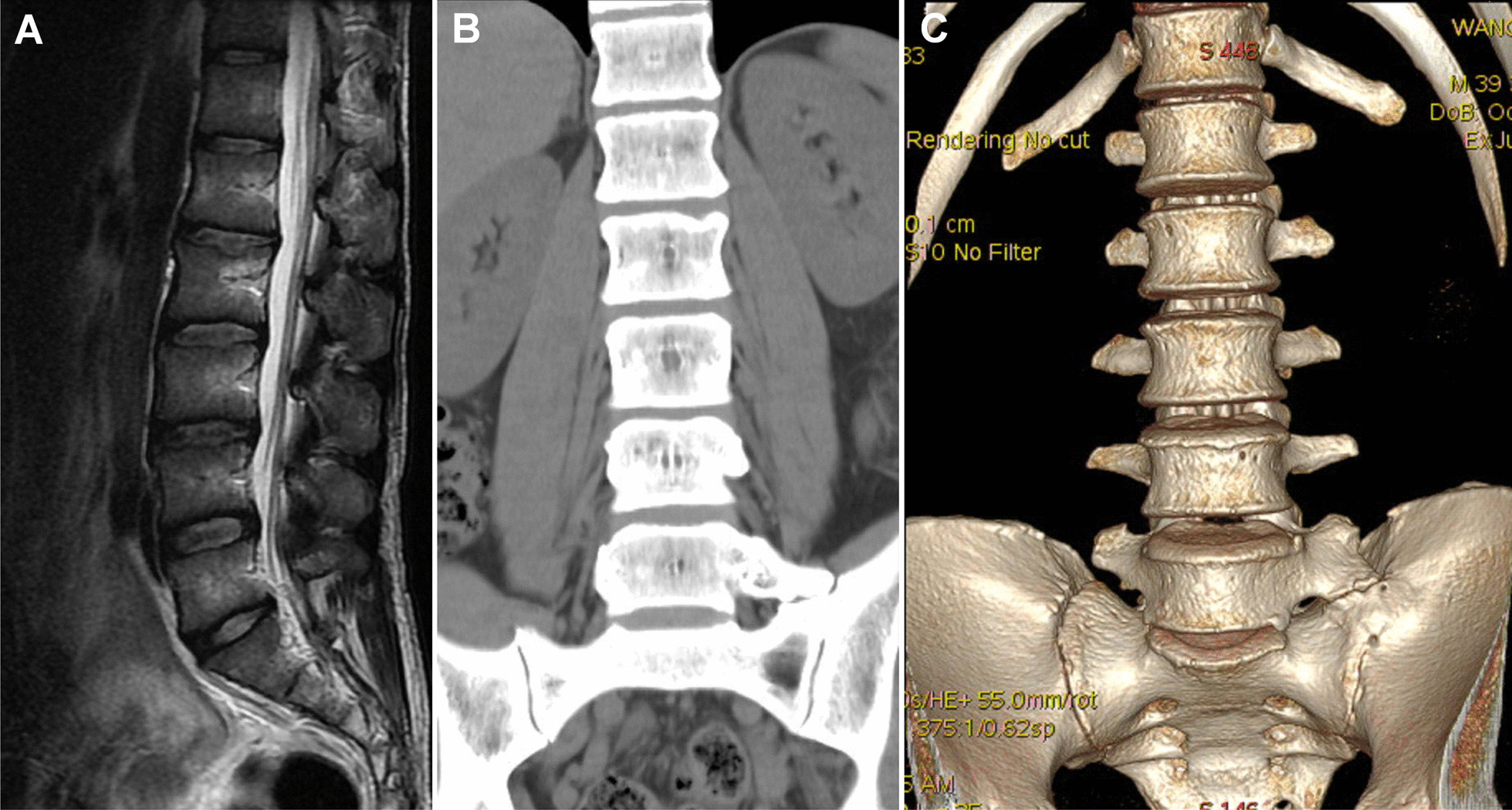
**A** Sagittal T2-weighted magnetic resonance image demonstrating only mild disc herniation, without evidence of foraminal stenosis. **B** Coronal computed tomography (CT) image taken before surgery, showing the anomalous enlargement of the left L5 transverse process, articulated with the left sacrum and ilium (Castellvi IIA). **C** Three-dimensional CT scan demonstrating the exact configuration of the extraforaminal stenosis at the anterior exit zone of the nerve root

A 39-year-old male individual presented with LBP and soreness lasting longer than 10 years. The symptoms included LBP that radiated from the back to the left buttock, thigh, lower leg, and foot area, which deteriorated after a full day of working primarily in the seated position, with long-distance walking, or after exercise. The pain was occasionally associated with local swelling and heat, electric shock–like tingling sensations, and toe numbness. The patient had previously received conservative treatments, such as medication, rehabilitation, local steroid injections, and acupuncture at another medical center without permanent symptomatic relief. Some doctors had previously advised the patient to undergo L4–5 discectomy, either with or without cage fusion, and the patient came to our hospital for seeking a second opinion. After admission, physical and neurological examinations revealed normal sensation, muscle power, and tendon reflexes in the lower limbs, with moderate local tenderness in the lower left back. The straight leg raising test (SLRT) was positive at 20° on the left side. The pain intensity was described as an 8, based on a 10-cm visual analog scale (VAS). A lumbar spine magnetic resonance imaging (MRI) analysis showed a mild disc herniation in L4–5 (Fig. 1A). Furthermore, lumbar computed tomography (CT) revealed the anomalous enlargement of the left L5 transverse process, which was articulated with the left sacrum and ilium, which was defined as an LSTV Type IIa according to the Castellvi classification system (Fig. 1B). A reconstructed three-dimensional CT image demonstrated extraforaminal stenosis, which might have caused left L5 nerve root impingement (Fig. 1C). Temporary pain relief was achieved following a local steroid injection near the left LSTV, resulting in the confirmation of a BS diagnosis.

**Fig. 2:**
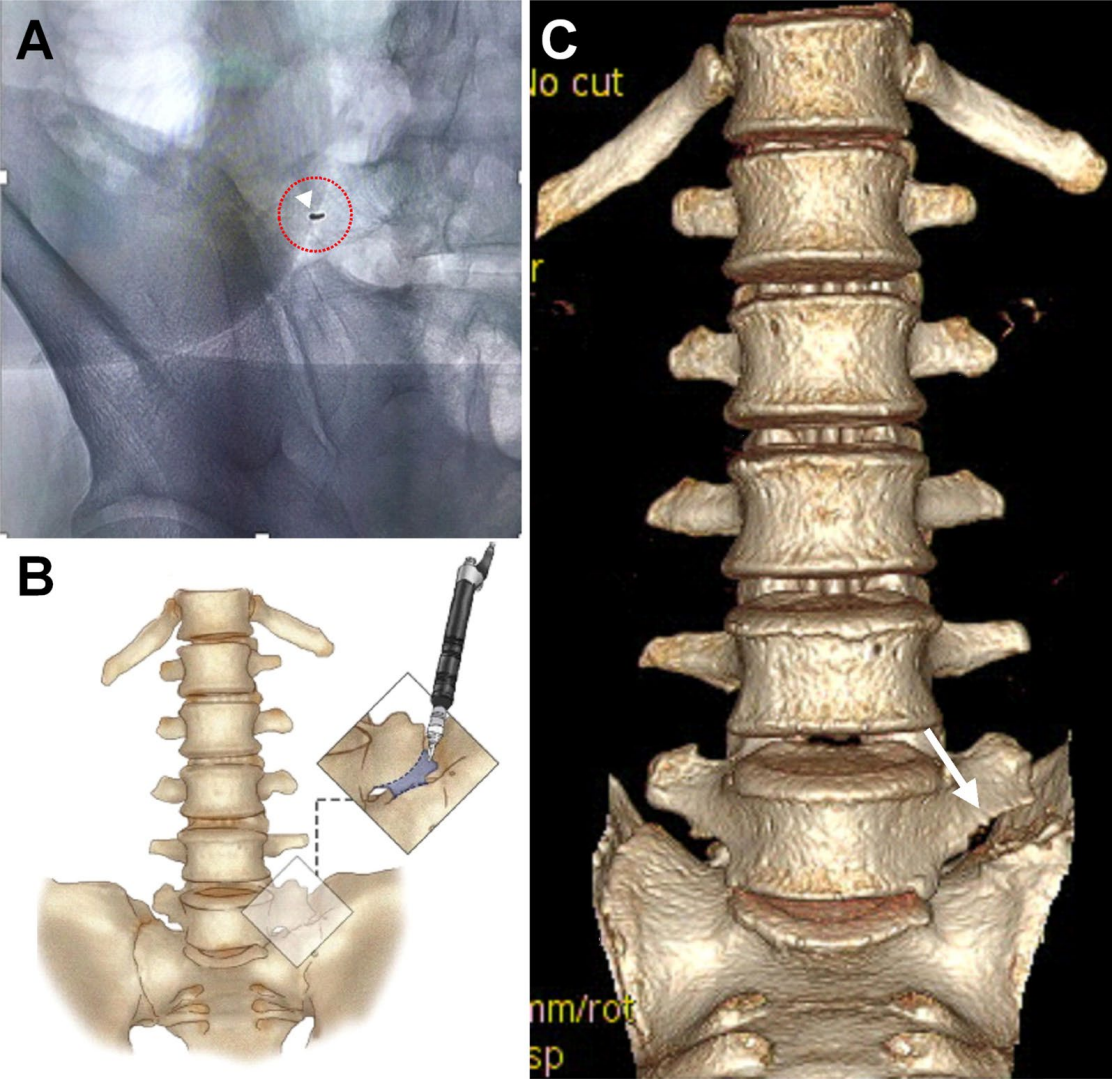
**A** Under fluoroscopy, a guide pin (arrowhead) was used to locate the pseudoarticulation. Centered on the guide pin, serial tubal dilators were inserted through the guide pin to enlarge the working port (red-dotted circle). **B** A schematic diagram showing the bone CUSA procedure for the neuroforaminal decompression, articulation dissection, and removal of the involved osteophytes. **C** Three-dimensional computed tomography scan demonstrating the post-operative lumbosacral transitional vertebrae articulation separation (arrow)

The patient underwent minimally invasive microscopic tubular articular resection (MISTAR) with the assistance of intraoperative three-dimensional C-arm image guidance. A guide pin was placed aiming to the articulation between the lumbar transverse process and the sacrum (Fig. 2A). Subsequently, a small, vertical skin incision centered the guide pin for approximately 3-cm off midline was made just above the posterior superior iliac spine. Serial tubal dilators were placed using the guide pin to enlarge the working port for LSTV exposure. To decompress the L5 nerve root and to open the neuroforamen, the involved osteophytes and iliolumbar ligamentous attachments were meticulously removed using a Kerrison Rongeur, a micro-curette, high-speed drills, and bone CUSA under microscopic guidance. After surgery, the complete disarticulation of the left L5 transverse process and the sacral alar were achieved (Fig. 2B). The L5 nerve root impingement and the opening of the neuroforamen exit zone were also relieved (Fig. 2C). The symptoms improved soon after the operation. The patient remained symptom-free without requiring the use of pain killers or additional therapies, and no interval changes or re-articulation were reported for up to 2 years of follow-up.

## Discussion and conclusion

LBP resulting from BS can have various etiologies, including (1) degeneration of the anomalous articulation, (2) facet joint arthrosis caused by unbalanced stress, (3) extraforaminal entrapment of nerve roots by the enlarged transverse process, and (4) early spondylolysis or spondylolisthesis at the level above the LSTV [5–8]. The simple resection of the pseudoarticulation is most effective in patients with degeneration of the anomalous articulation or facet joint pain above the LSTV. Decompression of the nerve root appears to be a suitable technique for patients with nerve root entrapment that induces radicular pain or for those who respond positively to a selective nerve root–block procedure. Fusion may be indicated in patients with LSTV co-existed with increased motion, instability, and adjacent segment problems [2].

**Fig. 3:**
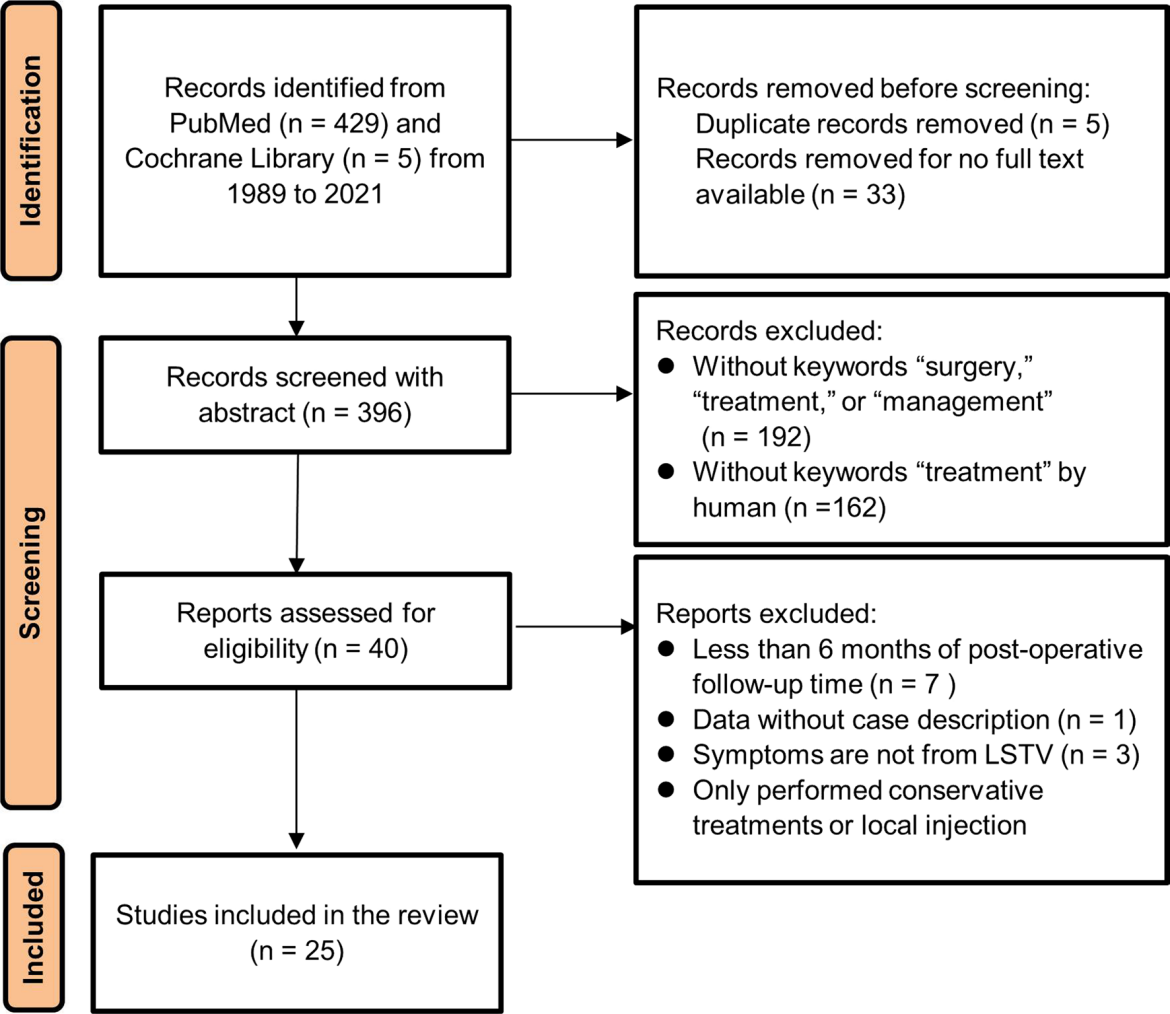
A flowchart presenting the literature review and analysis process

To review the long-term outcomes of BS treatments published in recent years, a rigeorous literature review was conducted based on the Preferred Reporting Items for Systematic Reviews and Meta-Analyses statement (Fig. 3) [15]. We searched PubMed and Cochrane Library from 1989 to 2021 using the keywords “Bertolotti syndrome” and “lumbosacral transitional vertebra,” which resulted in identifying 434 articles. Abstracts of these articles were further screened, excluding articles which did not contain the keywords “surgery,” “treatment,” or “management,”; did not provide case description; did not treat LSTV as the origin of the symptoms; or did not receive surgery. As a result, 25 publications describing 138 reported cases were extracted (Table 1). All articles described the surgical treatments of BS and included follow-up periods longer than 6 months with reported outcome evaluation data. To define unsatisfactory surgical outcomes, we assessed all cases with fair or poor postoperative results relative to the original assessments (fair or no improvement in VAS, Oswestry pain scores, Japanese Orthopedic Association scale, and Macnab criteria), and 20 cases from 4 publications were identified (Table 2).

**Table 1:** A review of surgical treatments for Bertolli’s syndrome with follow-up periods longer than 6 months

Intervention	Total cases	Age (y)	Type of LSTV^a^	Follow-up period (months)	Study type	References
Pulsed radiofrequency denervation	4	51.0	II(A): 3 II(B): 1	11.2	Case report	Ryo Kanematsu (2020); Burnham (2010) [10]
OMRDP	97	48.2	I: 21 II: 67 III: 4 Normal: 5	19.6	Case series (3) Case report (12)	Jönsson (1989) [14]; Santavirta (1993) [12]; Jeffrey (2001); Miyoshi (2011); Ichihara (2004); Almeida (2009); Cuenca (2019); Babu (2017); Ugokwe (2008); Louie (2019); Reitsma (2002); Son (2021); Ju (2017); Weber (2011); Dhanjani (2021); Iwasaki (2017)
OMRDA	5	47.2	II(A): 4 II(B): 1	16.8	Case report	Kikuchi (2013) [17]; Malham (2013) [18]; Abe (1997) [20]
MIS microscopic tubular resection	8	43.6	II(A): 2	22.6	Case series Case report	Shibayama (2011) [11]; Li (2014) [19]
Endoscopic decompression	14	59.5	–	11	Case series	Heo (2019) [16]
Fusion	10	34.5	II(A): 8 III(A): 1 III(B): 1	94.2	Case series Case report (2)	Santavirta (1993) [12]; Ise (2016); Dhanjani (2021)

*LSTV* lumbosacral transitional vertebrae, *OMRDP* open microscopic, articulation resection or nerve decompression via posterior approach, *MIS* minimally invasive surgery, *OMRDA* open microscopic, articulation resection or nerve decompression via anterior approach

^a^Castellvi classification; not all cases were classified in the included studies

**Table 2:** Reported unsatisfactory cases of Bertolli’s syndrome

Number of unsatisfactory cases/total cases	Intervention	Average age (y)	Type of LSTV	Neoarthrosis side (Uni- or Bilateral)	Response to local anesthesia	Outcome scale	Evaluation^a^			References
							E/G	F	P	
4/14	Endoscopic decompression	59.5	–	–	Yes	VAS, ODI, MacNab	10	3	1	Heo (2019) [16]
2/11 4/8 8/61	OMRDP	38.6 34.4 55.9	II(A) II(A) I–III	Uni Uni Uni: 12, Bi: 40	Yes Yes: 2, No:2 No, in 2 fair cases	– ODI VAS, MacNab	9 4 53	0 3 6	2 1 2	Jönsson (1989) [14]; Santavirta (1993) [12]; Ju (2017) [13]
2/8	Fusion	32.6	II(A): 7 III(B): 1	Uni: 7 Bi: 1	–	–	6	0	2	Santavirta (1993) [12]

*LSTV* lumbosacral transitional vertebrae, *VAS* visual analog scale, *ODI* Oswestry Disability Index, *OMRDP* open microscopic, articulation resection or nerve decompression via posterior approach

^a^Evaluation of outcomes are classified into excellent (E)/good (G), fair (F), and poor (P)

Most patients presented with good results after the surgery (118 of 138). However, some fair or poor results were reported (20 patients: 12 fair and 8 poor). Among the reported patients with unsatisfactory results, 14 received microscopic surgery (posterior approach for articulation resection or nerve root decompression) [12–14], 4 received endoscopic extraforaminal decompression [16], and 2 received posterior fusion [12]. Jönsson et al. reported 2 unfavorable cases who underwent resections of the transverse process and articulation [14]. The unfavorable outcome in one case might be the result of misdiagnosis, whereas no explanation was provided for the second case. Santavirta et al. reported 6 unfavorable cases in 1993; however, 4 of these cases did not receive or were not responsive to local diagnostic injections prior to surgical intervention [12]. Therefore, the origins of the pain were not clearly verified. Ju et al. reported 8 unfavorable cases and stated that the L5 transverse processectomy might contribute to the variousity of results according to different types of LSTV [13]. They described that L5 transverse processectomy as being significantly effective for Castellvi Type I and short joint–type Castellvi Type II patients; however, L5 transverse processectomy was not effective in wide joint–type of Castellvi Type II patients. These findings indicated that the correct and precise clinical or radiological diagnosis of the LSTV is essential to the achievement of satisfactory results. In addition, all unsatisfactory cases reported were performed via a posterior but not anterior approach. This outcome cannot be translated that the anterior is superior to posterior approach but may be due to the limited number of studies reporting the anterior approach [17, 18]. For these unsatisfactory open or microscopic posterior resection, common postoperative complications, such as L5 dermatome pain and development of a hematoma at the incision site were reported [19]. Despite no description of unsatisfactory outcomes associated with the anterior approach, one patient experienced neurapraxia in the left genitofemoral nerve, and another patient developed L5 radiculopathy after operation [18, 20].

Two out of eight patients who received fusion surgery reported unsatisfactory results [12]. However, this finding does not indicate that articulation resection alone is adequate, as most fusion patients are also associated with instability, and the creation of a larger open field might result in undesirable results. Santavirta et al. reported that 5 out of 8 patients who underwent articulation resections suffered from identifiable adjacent segment disc degeneration, whereas 7 of 8 patients experienced similar problems after fusion surgery [12]. In addition, the average follow-up period for the resection-only group (most of which were fewer than 3 years) was shorter than that of the fusion group (8.5 years). Therefore, conclusions regarding treatment superiority are difficult to derive because the resection-only group may develop additional problems after longer follow-up periods leading to dissatisfaction. These results indicate that the surgical decision for LSTV should be as comprehensive as possible, especially evaluations of the condition of adjacent disc and facet joints. These prudent considerations will result in good long-term results.

Endoscopic surgery appears to be associated with unsatisfactory results (28.5%, 4/14), according to a current report [16]. This outcome may be due to the unfamiliarity associated with the rarity of BS and distinct, individualized differences in the LSTV as successful endoscopic surgery is typically associated with a learning curve. In addition, undesirable endoscopic complications were reported, such as peri-renal fluid accumulation.

In our case, we only used cautery in the initial approach from the skin to the muscle, then applied a dissector, punch, and high-speed drill for the resection of the pseudoarticulation, and use bipolar and bone wax for hemostasis. We also used CUSA for the transverse process and neuroforamen decompression, which can prevent thermal injury and reduce injury to the muscular and ligamentous attachments surrounding the transverse process. However, there are still limitations when performing MISTAR. Firstly, adequate nerve decompression and de-articulation may be achieved in lieu of managing instability issue. Secondly, the narrow tubular working space may contribute to visual impairment which may result in further access difficulty and inadequate decompression. Thirdly, due to heavy scaring and an obscure surgical plane, it is difficult to apply the method to redo patients.. To sum up, no single operative method appears superior to any other method. Surgeons should make a clear diagnosis, confirm every anatomical detail, choose a familiar technique, and develop an overall and foreseeing surgical plan to achieve adequate decompression and prevent unnecessary tissue injury when they encounter this rare disease.

The present report suggests that minimally invasive tubular microscopic decompression and disarticulation with the assistance of a navigator and CUSA can be used as an effective surgical treatment for BS characterized by LSTV. However, assessing anatomical variations and individualized differences are the keys to achieving successful outcomes.

## Data Availability

The datasets used and/or analyzed in the current study are available from the corresponding author upon reasonable request.

## Abbreviations

LBP: Low back pain
LSTV: Lumbosacral transitional vertebrae
BS: Bertolotti’s syndrome
CUSA: Cavitron ultrasonic surgical aspirator
SLRT: Straight leg raising test
VAS: Visual analog scale
MRI: Magnetic resonance imaging
CT: Computed tomography
MISTAR: Minimally invasive microscopic tubular articular resection

## References

[1] Jancuska JM, Spivak JM, Bendo JA (2015). A review of symptomatic lumbosacral transitional vertebrae: Bertolotti’s syndrome. Int J Spine Surg.

[2] McGrath K, Schmidt E, Rabah N, Abubakr M, Steinmetz M. Clinical assessment and management of Bertolotti Syndrome: a review of the literature. Spine J. 2021. 10.1016/j.spinee.2021.02.023 33676018

[3] Quinlan JF, Duke D, Eustace S (2006). Bertolotti’s syndrome. A cause of back pain in young people. J Bone Joint Surg Br.

[4] Castellvi AE, Goldstein LA, Chan DP (1984). Lumbosacral transitional vertebrae and their relationship with lumbar extradural defects. Spine.

[5] Paik NC, Lim CS, Jang HS (2013). Numeric and morphological verification of lumbosacral segments in 8280 consecutive patients. Spine.

[6] Otani K, Konno S, Kikuchi S (2001). Lumbosacral transitional vertebrae and nerve-root symptoms. J Bone Joint Surg Br.

[7] Luoma K, Vehmas T, Raininko R, Luukkonen R, Riihimaki H (2004). Lumbosacral transitional vertebra: relation to disc degeneration and low back pain. Spine.

[8] Holm EK, Bunger C, Foldager CB (2017). Symptomatic lumbosacral transitional vertebra: a review of the current literature and clinical outcomes following steroid injection or surgical intervention. SICOT J.

[9] Marks RC, Thulbourne T (1991). Infiltration of anomalous lumbosacral articulations. Steroid and anesthetic injections in 10 back-pain patients. Acta Orthop Scand.

[10] Burnham R (2010). Radiofrequency sensory ablation as a treatment for symptomatic unilateral lumbosacral junction pseudarticulation (Bertolotti’s syndrome): a case report. Pain Med.

[11] Shibayama M, Ito F, Miura Y, Nakamura S, Ikeda S, Fujiwara K (2011). Unsuspected reason for sciatica in Bertolotti’s syndrome. J Bone Joint Surg Br.

[12] Santavirta S, Tallroth K, Ylinen P, Suoranta H (1993). Surgical treatment of Bertolotti’s syndrome. Follow-up of 16 patients. Arch Orthop Trauma Surg.

[13] Ju CI, Kim SW, Kim JG, Lee SM, Shin H, Lee HY (2017). Decompressive L5 transverse processectomy for Bertolotti’s syndrome: a preliminary study. Pain Physician.

[14] Jonsson B, Stromqvist B, Egund N (1989). Anomalous lumbosacral articulations and low-back pain. Evaluation and treatment. Spine.

[15] Page MJ, McKenzie JE, Bossuyt PM, Boutron I, Hoffmann TC, Mulrow CD, Shamseer L, Tetzlaff JM, Akl EA, Brennan SE, Chou R, Glanville J, Grimshaw JM, Hrobjartsson A, Lalu MM, Li T, Loder EW, Mayo-Wilson E, McDonald S, McGuinness LA, Stewart LA, Thomas J, Tricco AC, Welch VA, Whiting P, Moher D (2021). The PRISMA 2020 statement: an updated guideline for reporting systematic reviews. BMJ.

[16] Heo DH, Sharma S, Park CK (2019). Endoscopic treatment of extraforaminal entrapment of L5 nerve root (far out syndrome) by unilateral biportal endoscopic approach: technical report and preliminary clinical results. Neurospine.

[17] Kikuchi K, Abe E, Miyakoshi N, Kobayashi T, Abe T, Hongo M, Shimada Y (2013). Anterior decompression for far-out syndrome below a transitional vertebra: a report of two cases. Spine J.

[18] Malham GM, Limb RJ, Claydon MH, Brazenor GA (2013). Anterior pseudoarthrectomy for symptomatic Bertolotti’s syndrome. J Clin Neurosci.

[19] Li Y, Lubelski D, Abdullah KG, Mroz TE, Steinmetz MP (2014). Minimally invasive tubular resection of the anomalous transverse process in patients with Bertolotti’s syndrome: presented at the 2013 Joint Spine Section Meeting: clinical article. J Neurosurg Spine.

[20] Abe E, Sato K, Shimada Y, Okada K, Yan K, Mizutani Y (1997). Anterior decompression of foraminal stenosis below a lumbosacral transitional vertebra. A case report. Spine.

